# Effects of new antiseizure medication on bone metabolism and bone mineral density in children: A meta-analysis

**DOI:** 10.3389/fped.2022.1015691

**Published:** 2022-11-15

**Authors:** La Zhuo, Yong Zhang

**Affiliations:** ^1^ Department of Pharmacy, Maternal and Child Health Hospital of Inner Mongolia Autonomous Region, Hohhot City, China; ^2^ Department of Clinical Pharmacy, Children's Hospital of Nanjing Medical University, Nanjing, China

**Keywords:** children, bone mineral density, bone metabolism, pediatrics, new antiseizure medication

## Abstract

**Objective:**

To explore the effect of new antiseizure medication on bone metabolism and bone mineral density in children**.**

**Methods:**

The Chinese and English databases (PubMed, EMBASE, Cochrane Library, CNKI, Wanfang and VIP) were systematically searched for observational studies evaluating the effects of new antiseizure medication on bone metabolism and bone mineral density in children with epilepsy. The effects of new antiseizure medication on serum calcium, phosphorus, alkaline phosphatase, bone alkaline phosphatase, parathyroid hormone, 25-hydroxyvitamin D and bone mineral density in children were systematically evaluated.

**Results:**

After systematic retrieval and screening, 12 studies with high literature quality (including 629 epileptic children and 627 control subjects) were included in the systematic evaluation. Meta-analysis showed that new antiseizure medication decreased bone mineral density (MD: −0.05, 95%CI, −0.09, −0.02; *P *= 0.004). From different kinds of antiseizure medication, levetiracetam can reduce blood phosphorus concentration in children (MD: −0.04; 95%CI, −0.07, −0.01). Oxcarbazepine increased serum alkaline phosphatase in children (MD:17.98; 95%CI, 10.43,25.53; *P *< 0.00001), and the increase intensity was significantly higher than that of levetiracetam (MD: 7.66; 95%CI, 0.29, 15.02; *P *= 0.04). In addition, oxcarbazepine can cause a significant increase in parathyroid hormone in children (MD: 7.52; 95%CI, 3.37,11.66; *P *= 0.0004), and 25 - hydroxyvitamin D was reduced, and the difference was statistically significant (MD:−2.18; 95%CI, −3.23, −1.13; *P *= 0.00006). However, the effects of new antiseizure medication on serum calcium and bone alkaline phosphatase in children were not statistically significant

**Conclusion:**

New antiseizure medication have different effects on bone metabolism and bone mineral density in children with epilepsy, and the effects of different types of new antiseizure medication are different.

## Introduction

Epilepsy is one of the most common neurological diseases, affecting about 50 million people worldwide (1). Epidemiological survey data show that the possibility of a seizure in one lifetime is 10%, and the annual incidence of epilepsy is 50.4–81.7/100,000 (2). Among common epilepsy and children, 5–9 years old is the peak of epilepsy in children (3). There are significant changes in growth with an approximately threefold increase in length, dramatic changes in the relative proportions of limb, body, and head size, and the accumulation of large amounts of calcium and phosphate within the bones, from birth to the end of pubertal growth (4). At present, the use of antiseizure medication is an effective measure to control the recurrence of epilepsy, which can control 70% of patients with epilepsy (5).

However, since the 1960s, it has been reported that antiseizure medication can lead to bone disease (6), more and more scholars began to pay attention to the effect of antiseizure medication on bone metabolism. Early reports describe a significantly increased risk of hypocalcemia, radiologic evidence of rickets, decreased bone mineral density, and osteomalacia, particularly in subjects treated with antiseizure medication polytherapy (7, 8). Kumandas et al. (9) believed that carbamazepine as a liver enzyme inducer could reduce the bone mineral density of children. Babayigit et al. (10) found that valproic acid as a non-liver enzyme inducer could also reduce the bone mineral density of children. Compared with traditional antiseizure medication, new antiseizure medication has great improvement in adverse reactions and drug interactions, so new antiseizure medication is widely used in clinical practice. Whether new antiseizure medication affects bone metabolism has attracted more and more attention. As a member of new antiseizure medication, oxcarbazepine has a weak induction effect on liver enzymes. Mintzer et al. (11) found that oxcarbazepine could reduce serum vitamin D in patients with epilepsy, while Jin et al. (12) did not find the effect of oxcarbazepine on bone turnover markers and bone mineral density in children. Although some studies have documented the effects of new antiseizure medication on bone mineral density and bone metabolism in children, there is still no consistent report.

Therefore, the purpose of this study is to integrate the current data and explore the effect of new antiseizure medication on bone mineral density and bone metabolism in children with epilepsy, so as to provide high-quality evidence for the health management of children with epilepsy.

## Materials and methods

### Search strategy

Following the PRISMA guidebook, A systematic literature search of PubMed, Embase, Cochrane Library, Web of Science, CINAHL, CNKI, Wanfang database and VIP database was performed from the date of inception of the databases to June 30, 2022. The search terms included Children, Epilepsy, Bone mineral density, Bone metabolism, antiseizure medication, Ocarbazepine, Topiramate, Levetiracetam, Lamotrigine, Clobazam, Gabapentin. Synonyms of each term were also used.

### Inclusion of exclusion criteria

Inclusion criteria: (1) Chinese and English studies published in peer review journals and included only Chinese studies published in core journals; (2) Subjects were diagnosed with epilepsy in children, age <18 years old; (3) Treatment with new antiseizure medication; (4) The control group was healthy people without drug intervention or epilepsy children without antiseizure medication or self-control; (5) At least one bone mineral density or bone metabolism index was included after treatment; (6) Research types include observational studies and randomized controlled trials. Exclusion criteria: (1) Non-population studies; (2) Conference articles, case reports, systematic reviews and other research types; (3) The outcome information is insufficient and cannot be analyzed; (4) Repeated reports of literature research. (5) The study of complete articles cannot be obtained.

### Study selection and data extraction

Two reviewers independently reviewed the abstracts and full text of each article acorrding to inclusion of exclusion criteria. For disagreements between the two reviewers, a third reviewer was recruited for discussion until consensus was achieved. After literature screening, two reviewers independently respectivly extracted the following information: Literature information, demographic characteristics of the subjects, and the information of dosage, duration and outcome indicators of new antiseizure medication.

### Assessments of quality

Newcastle-Ottawa Scale (NOS) was used to evaluate the quality of observational studies. The scale was evaluated from eight aspects, including the representativeness of the research population, the comparability between groups, whether the evaluation of the results was sufficient, whether the follow-up time was sufficient, and the integrity of the follow-up. The full score was 9 points. The total score was 7 points and above for high-quality literature, and 5 points and below for low-quality articles.

### Statistical analysis

Revman 5.3 software was used for statistical analysis. The effect of measurement data is represented by the relative risk weighted mean difference (MD), and the 95% confidence interval (CI) is used to estimate the interval range of the effect. Heterogeneity test was used to determine the size of heterogeneity by the test of $I^{2}$. if $I^{2}<50\%$ or *P* > 0.1, it was considered that the included literature was homogeneous, and the fixed effect model (Mantel-Haenszel) was used for analysis; if $I^{2}>50\%$ or *P *≤ 0.1, it was considered that the included studies were heterogeneous, and the random effect model (DerSimonian-Laird) was used for analysis. If heterogeneity was large, sensitivity analysis was used to explore the source of heterogeneity. *P *< 0.05 indicated that the difference was statistically significant.

## Results

### Basic characteristics and literature quality evaluation of included studies

**Figure 1:**
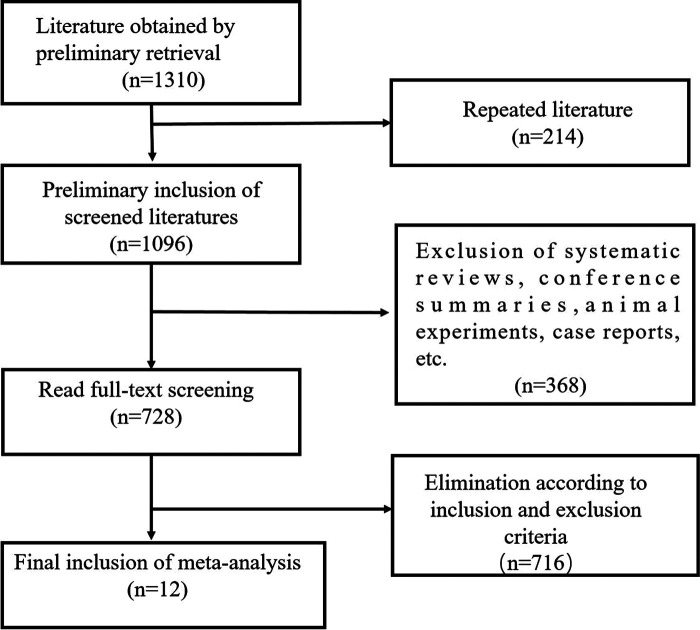
Flow chart of target literature screening.

After systematic search and screening of Chinese and English databases, 12 eligible studies (10, 12–22) were included in this study. The flow chart of literature screening is shown in Figure 1. Twelve studies involved 629 children with epilepsy and 627 controls. The control group of seven studies was healthy children, the control group of two studies was children without antiseizure medication, and the other three studies were self-control. Seven studies used oxcarbazepine, four studies used levetiracetam, four studies used topiramate, and three studies used lamotrigine. The results of literature quality evaluation showed that the quality of included studies was high, and the average NOS score was 7.3. The basic characteristics of more included studies are shown in Table 1.

**Table 1:** Basic characteristics of included studies and literature quality evaluation results table

Study (Author, year)	country	study design	sample size (O/C)	Years (O/C)	male (O/C)	control group	Drugs	dosage	duration	NOS
Zhao, 2020	China	retrospective cohort	20/20	7.70 ± 2.96/8.34 ± 2.16	12/11	healthy children	OXC	10–40 mg/kg/d	12 months	8
Zhang, 2020a	China	prospective cohort	50/50	9.56 ± 1.28	34	self-control	OXC	20–30 mg/kg/d	6 months	8
Zhang, 2020b	China	prospective cohort	50/50	9.47 ± 1.32	35	self-control	LEV	20–30 mg/kg/d	6 months	
Zhang, 2012	China	retrospective cohort	40/25	3–12	21/NR	healthy children	TPM	3–5 mg/kg/d	3 months	6
Zhang, 2010	China	retrospective cohort	32/36	10.1 ± 2.4/9.1 ± 3.0	NR	patients without drugs	TPM	3–5 mg/kg/d	0.5–1.5 years	7
Yan, 2020a	China	prospective cohort	40/40	8.2 ± 2.49	21	self-control	OXC	NR	12 months	8
Yan, 2020b	China	prospective cohort	40/40	7.97 ± 2.54	18	self-control	TPM	NR	12 months	
Yan, 2020c	China	prospective cohort	40/40	7.90 ± 2.53	20	self-control	LTG	NR	12 months	
Yan, 2020d	China	prospective cohort	40/40	8.10 ± 2.48	19	self-control	LEV	NR	12 months	
Arzu, 2006	Turkey	retrospective cohort	14/30	13.13 ± 3.17/13.09 ± 3.09	5/14	healthy children	OXC	15–30 mg/kg/d	2.36 ± 0.86 years	8
Raj, 2007	USA	retrospective cohort	13/36	12.1 ± 3.9/12.9 ± 2.6	6/14	healthy children	LTG	NR	5 years	7
Markus, 2009	Austria	retrospective cohort	40/41	11.83 ± 3.0/12.08 ± 3.41	28/29	healthy children	LTG, OXC	NR	≥6 months	6
Babacan, 2012	Turkey	retrospective cohort	44/33	9.65 ± 3.04/10.24 ± 2.86	22/17	healthy children	OXC	NR	≥1 year	8
Serin, 2015	Turkey	retrospective cohort	20/20	8.15 ± 3.3/7.6 ± 3.3	14/13	healthy children	LEV	NR	≥2 years	8
Zhang, 2008	China	retrospective cohort	50/30	3–12	26/NR	patients without drugs	TPM	3–5 mg/kg/d	12 months	7
Jin, 2014a	China	retrospective cohort	42/42	8.7 ± 2.1	24	self-control	OXC	10–60 mg/kg/d	1 year	7
Jin, 2014b	China	retrospective cohort	54/54	8.9 ± 2.3	30	self-control	LEV	5–60 mg/kg/d	1 year	

O, observation group; C, control group; OXC, oxcarbazepine; LEV, levetiracetam; TPM, topiramate; LTG, lamotrigine; NR, not reported.

### Effect of new antiseizure medication on serum calcium in epileptic children

**Figure 2:**
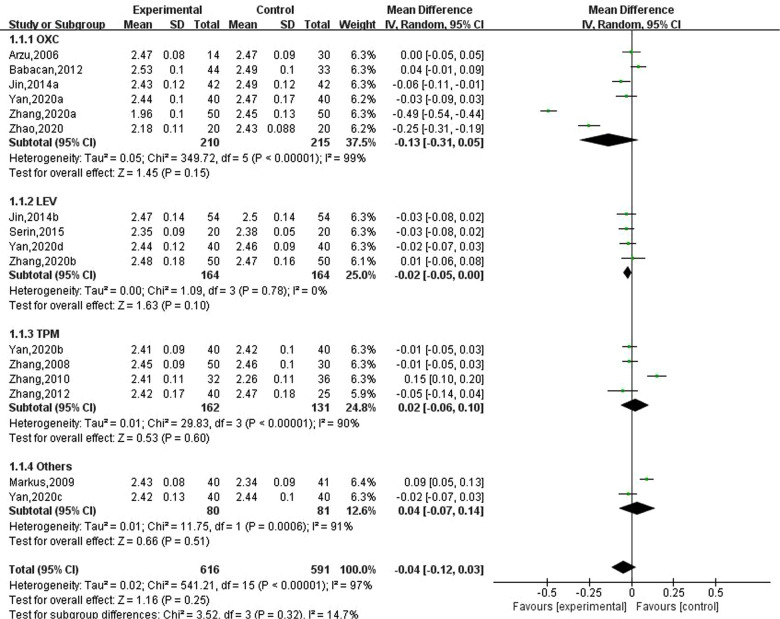
Subgroup analysis of the effect of new antiseizure medication on serum calcium (mmol/L) in children with epilepsy.

Eleven studies reported the effect of new antiseizure medication on serum calcium in children with epilepsy. A total of 616 children took antiseizure medication and 591 control subjects. Six studies used oxcarbazepine, four studies used levetiracetam and topiramate, one study used lamotrigine, and another study evaluated the effects of oxcarbazepine and lamotrigine. Heterogeneity evaluation results showed that there was significant heterogeneity among the included studies (*P* < 0.00001). Random effect model was used and subgroup analysis was performed according to different drugs. The results of systematic evaluation showed that compared with the subjects without antiseizure medication, the effects of oxcarbazepine, levetiracetam and topiramate on serum calcium in children with epilepsy were not statistically significant, and the effects were −0.13 (95%CI, −0.31, 0.05), −0.02 (95%CI, −0.05, 0.00) and 0.02 (95%CI, −0.06, 0.10), respectively. In terms of the final combined effect, the use of new antiseizure medication has a tendency to reduce the serum calcium content in children with epilepsy, as shown in Figure 2. Sensitivity analysis showed no significant heterogeneity, suggesting that the heterogeneity of included studies was stable.

### Effect of new antiseizure medication on serum phosphorus in epileptic children

**Figure 3:**
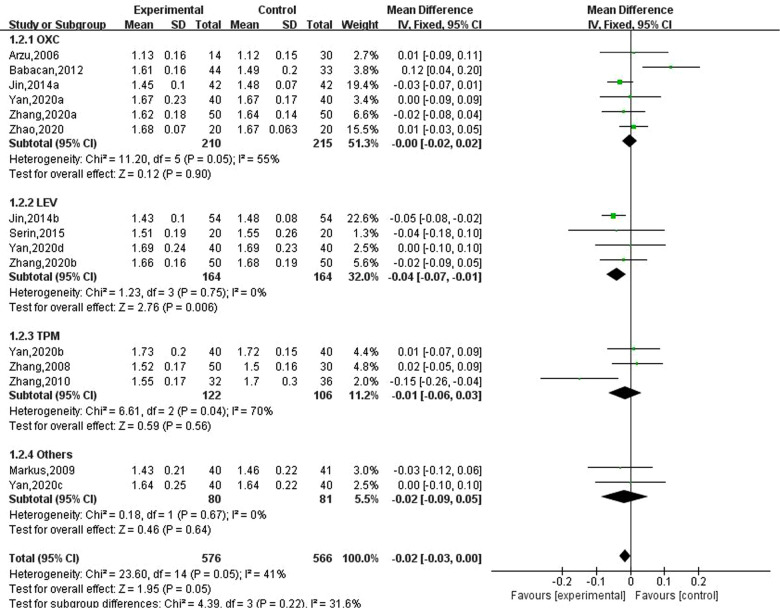
Subgroup analysis of the effect of new antiseizure medication on serum phosphorus (mmol/L) in epileptic children.

Ten studies involving 15 data reported the results of serum phosphorus in children with epilepsy after taking antiseizure medication, of which 576 children with epilepsy took new antiseizure medication and a total of 566 subjects in the control group. Six studies used oxcarbazepine, four used levetiracetam, three used topiramate, one used lamotrigine, and the other evaluated the effect of oxcarbazepine and lamotrigine. The heterogeneity evaluation showed that there was little heterogeneity among the included studies ($I^{2}=41\%$, *P *= 0.05), and the fixed effects model was used to calculate the combined effect size. Meta-analysis showed that serum phosphorus concentrations were decreased by 0.02 mmol/L (95% CI, −0.03, 0.00; *P *= 0.05) in epileptic children after taking novel antiseizure medication. Subgroup analysis of different classes of novel antiseizure medication showed that levetiracetam significantly reduced serum phosphorus concentrations in children (MD: −0.04; 95% CI, −0.07, −0.01), however, oxcarbazepine and topiramate did not have a statistically significant effect in reducing serum phosphorus in children compared to controls. See Figure 3.

### Effect of new antiseizure medication on serum alkaline phosphatase in epileptic children

**Figure 4:**
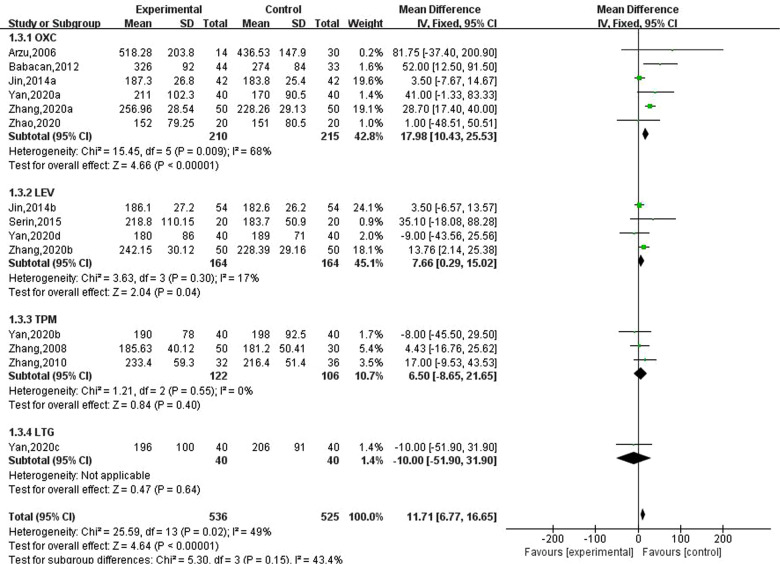
Subgroup analysis of the effect of new antiseizure medication on serum alkaline phosphatase (U/L) in epileptic children.

Nine studies involving 14 data reported the results of serum alkaline phosphatase in children with epilepsy after taking new antiseizure medication. Among them, 536 children took new antiseizure medication, and 525 controls did not take new antiseizure medication. Six studies used oxcarbazepine, four studies used levetiracetam, three studies used topiramate, and one study used lamotrigine. The heterogeneity evaluation results showed that the homogeneity among the included studies was good ($I^{2}=49\%$, *P* = 0.02), and the fixed effect model was used for systematic evaluation. Meta-analysis results showed that compared with the control group, the serum alkaline phosphatase of epileptic children was significantly increased after taking new antiseizure medication (MD: 11.71; 95%CI, 6. 77,16. 65; *P *< 0.00001). For different types of new antiseizure medication, oxcarbazepine had the greatest effect on serum alkaline phosphatase (MD: 17.98; 95%CI, 10.43,25.53; *P *< 0.00001), which was significantly higher than the influence intensity of Levetiracetam (MD: 7.66; 95%CI, 0. 29,15. 02; *P *= 0.04). However, after taking topiramate, the increase of serum alkaline phosphatase in children was not statistically significant (MD: 6.50; 95%CI, −8.65,21.65; *P *= 0.40). See Figure 4.

### Effect of new antiseizure medication on bone alkaline phosphatase in epileptic children

**Figure 5:**
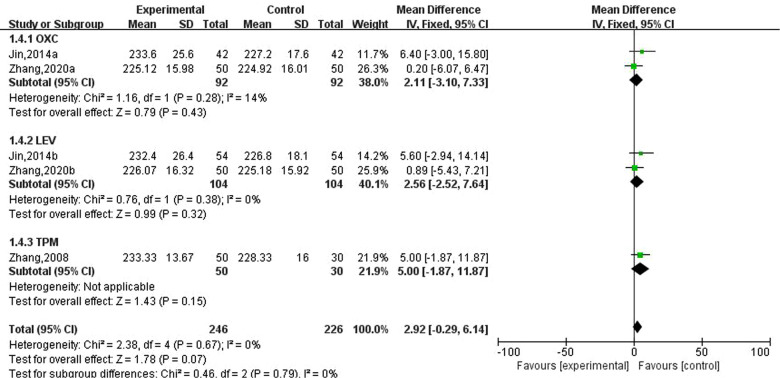
Subgroup analysis of the effect of new antiseizure medication on bone alkaline phosphatase (U/L) in epileptic children.

Three studies involving five data reported the effects of new antiseizure medication on bone alkaline phosphatase in epileptic children. Among them, 246 children took new antiseizure medication and 226 subjects in the control group. Two studies used oxcarbazepine and levetiracetam, and another study used topiramate. The heterogeneity evaluation results showed that there was a high homogeneity among the included studies ($I^{2}=0\%$, *P* = 0.67), and the fixed effect model was used for systematic evaluation. Meta-analysis results showed that the effects of oxcarbazepine, levetiracetam and topiramate on bone alkaline phosphatase in epileptic children were not statistically significant, as shown in Figure 5.

### Effect of new antiseizure medication on parathyroid hormone in children with epilepsy

**Figure 6:**
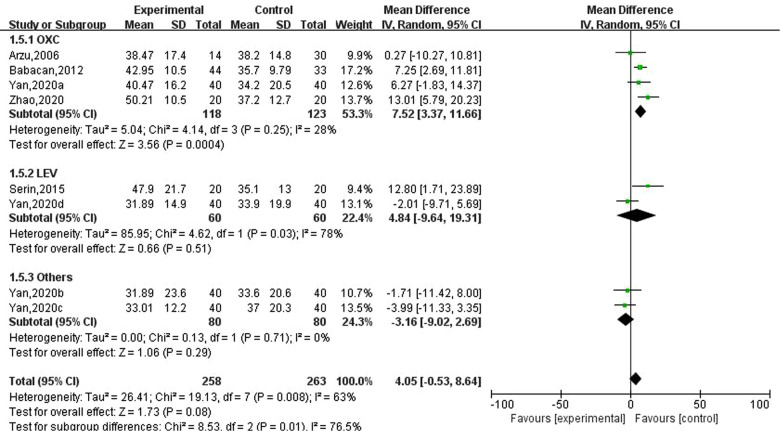
Subgroup analysis of the effect of new antiseizure medication on parathyroid hormone (pg/ml) in epileptic children.

Five studies involving eight data reported the effects of new antiseizure medication on thyroid hormones in children with epilepsy. Among them, 258 children took new antiseizure medication, and 263 subjects in the control group. Four studies used oxcarbazepine, two studies used levetiracetam, and one study used topiramate and lamotrigine, respectively. Heterogeneity evaluation results showed that there was a certain heterogeneity between the included studies ($I^{2}=63\%$, *P *= 0.008), and the random effect model was used to calculate the combined effect. Subgroup analysis showed that after taking oxcarbazepine, parathyroid hormone was significantly increased, and the difference was statistically significant (MD: 7.52; 95%CI, 3.37,11.66; *P *= 0.0004). But the effects of levetiracetam, topiramate and lamotrigine on thyroid hormones were not statistically significant, as shown in Figure 6. Sensitivity analysis did not find significant sources of heterogeneity, suggesting that the heterogeneity between included studies was relatively stable.

### Effect of new antiseizure medication on 25-hydroxyvitamin D in epileptic children

**Figure 7:**
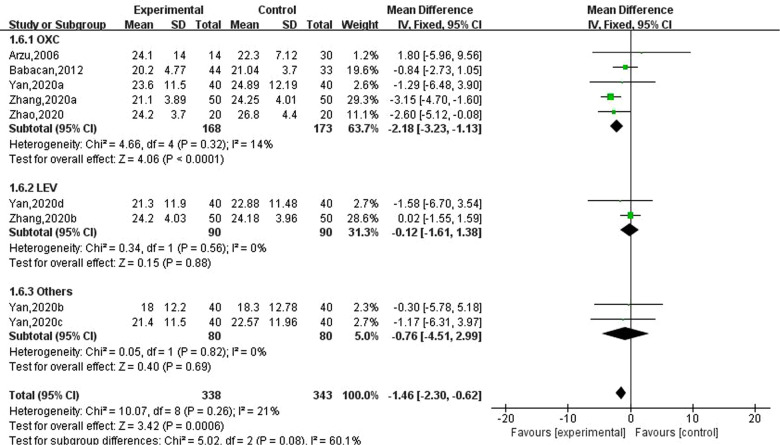
Subgroup analysis of the effect of new antiseizure medication on 25-hydroxyvitamin D (nmol/L) in epileptic children.

Five studies involving nine data reported the effects of new antiseizure medication on 25-hydroxyvitamin D in epileptic children, including 338 children in the observation group and 343 subjects in the control group. Five studies used oxcarbazepine, two studies used levetiracetam, and one study used topiramate and lamotrigine, respectively. The results of heterogeneity evaluation suggested that there was low heterogeneity among the included studies ($I^{2}=21\%$, *P *= 0.26), and the fixed effect model was used for systematic evaluation. The results showed that oxcarbazepine could reduce 25-hydroxyvitamin D in children with epilepsy, and the difference was statistically significant (MD: −2.18; 95%CI, −3.23, −1.13; *P *= 0.00006), the effects of levetiracetam, topiramate and lamotrigine on 25 - hydroxyvitamin D were not statistically significant. See Figure 7.

### Effect of new antiseizure medication on bone mineral density in children with epilepsy

**Figure 8:**
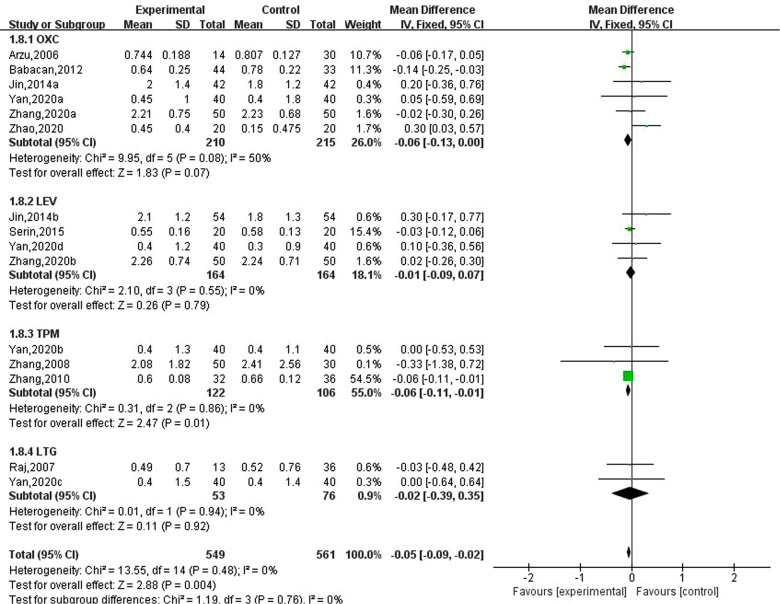
Subgroup analysis of the effect of new antiseizure medication on bone mineral density in children with epilepsy.

Ten studies involving 15 data reported the effect of new antiseizure medication on bone mineral density in children with epilepsy. Among them, 549 children took new antiseizure medication, and 561 subjects in the control group. Six studies used oxcarbazepine, four studies used levetiracetam, three studies used topiramate, and two studies used lamotrigine. The results of heterogeneity evaluation showed that the included studies had good homogeneity ($I^{2}=0\%$, *P *= 0.48), and the fixed effect model was used for systematic evaluation. The results showed that the new antiseizure medication reduced the bone mineral density of children (MD: −0.05, 95%CI, −0.09, −0.02; *P *= 0.004). From the results of different types of new antiseizure medication, oxcarbazepine and topiramate significantly reduced bone mineral density in children, and the difference was statistically significant. However, the effects of levetiracetam and lamotrigine on bone mineral density were not statistically significant. See Figure 8.

## Discussion

After systematic retrieval and screening of Chinese and English databases, 12 qualified studies were finally included in the final systematic evaluation. The results of systematic evaluation showed that the treatment of new antiseizure medication was related to the decrease of bone mineral density. In addition, oxcarbazepine and the increase of serum alkaline phosphatase and parathyroid hormone, and the decrease of 25-hydroxyvitamin D were related. Levetiracetam was associated with decreased serum phosphorus and increased serum alkaline phosphatase. However, this study did not find that the effect of new antiseizure medication on serum calcium and bone alkaline phosphatase in epileptic children was statistically significant.

Previous studies have shown that there is a correlation between long-term use of antiseizure medication and metabolic, endocrine and hormone disorders, even if the prescribed dose range of antiseizure medication is used (23–25). Patients with epilepsy are prone to bone health problems. On average, 50% of patients with epilepsy have bone abnormalities (26, 27), and the risk of fracture increases by 2–6 times (28–30). It is important to recognize that especially high-dose antiseizure medication may cause poor coordination which can increase fracture susceptibility (31). Sibling pair studies which compared measures of static and dynamic balance between siblings treated with antiseizure medication and siblings on no antiseizure medication showed that users of antiseizure medication performed significantly worse on the measures with longer duration of antiseizure medication therapy resulting in worse scores (32, 33).

The relationship between antiseizure medication and bone health in patients with epilepsy has been widely recognized. An explanation mechanism for the decrease of bone mineral density caused by antiseizure medication is that antiseizure medication induce the liver *P*-450 enzyme system, resulting in an increase in 25-hydroxyvitamin D catabolism, resulting in relatively hypocalcemia, an increase in parathyroid hormone level, and ultimately a decrease in bone mineral density (11, 34, 35). However, the exact mechanism remains to be further studied.

Although there are few available data on children, since childhood is a critical period for bone mass development, the bone health of children with epilepsy is particularly worthy of attention. Cansu et al. (36) found that patients treated with oxcarbazepine compared with healthy controls, alkaline phosphatase and parathyroid hormone increased significantly, 25-hydroxyvitamin D levels decreased. Babayigit et al. (10) reported that compared with healthy children, children treated with oxcarbazepine had higher alkaline phosphatase and lower bone mineral density, which was consistent with the results of this study. In addition, it is worth noting that previous studies have shown that children receiving additional antiseizure medication may have a higher risk of fracture than children receiving single therapy (37).

This study has some limitations. First of all, due to the differences in the length of the study and the age, sex ratio, epilepsies type and drug dose of the subjects, there may be some heterogeneity between the included studies, resulting in mixed effects. In addition, the sample size of included studies was small, and the sample size of two studies was less than 20, which may lead to potential bias among the study population. Since the current research on children in this field is relatively small, only 12 target literatures are included for evaluation in this study, and some outcome indicators are rarely included. Finally, the included studies were to explore the relationship between single drug use and bone health, and the effect of multi-drug treatment on bone mineral density and bone metabolism in children with epilepsy was lacking.

## Conclusion

In summary, the results of this study suggest that new antiseizure medication had a certain negative effect on bone mineral density and bone metabolism in children with epilepsy. In clinical practice, it is necessary to dynamically monitor the changes in bone mineral density and bone metabolism in children with epilepsy during medication, and give reasonable intervention measures to avoid the occurrence of adverse outcomes. In addition, due to the limitations of this study, more high-quality studies are needed in the future to further determine the impact of new antiseizure medication on children with epilepsy and clarify the relationship between drug type, drug dose, medication time and other factors and bone mineral density and bone metabolism.

## Data Availability

The original contributions presented in the study are included in the article/Supplementary Material, further inquiries can be directed to the corresponding author/s.
